# Development and Validation of an Explainable Deep Learning Model to Predict In-Hospital Mortality for Patients With Acute Myocardial Infarction: Algorithm Development and Validation Study

**DOI:** 10.2196/49848

**Published:** 2024-05-10

**Authors:** Puguang Xie, Hao Wang, Jun Xiao, Fan Xu, Jingyang Liu, Zihang Chen, Weijie Zhao, Siyu Hou, Dongdong Wu, Yu Ma, Jingjing Xiao

**Affiliations:** 1 Chongqing Emergency Medical Centre, Chongqing University Central Hospital, School of Medicine, Chongqing University Chongqing China; 2 Bioengineering College, Chongqing University Chongqing China; 3 Bio-Med Informatics Research Centre & Clinical Research Centre, Xinqiao Hospital, Army Medical University Chongqing China; 4 Medical Big Data Research Centre, Chinese People's Liberation Army General Hospital Beijing China

**Keywords:** acute myocardial infarction, mortality, deep learning, explainable model, prediction

## Abstract

**Background:**

Acute myocardial infarction (AMI) is one of the most severe cardiovascular diseases and is associated with a high risk of in-hospital mortality. However, the current deep learning models for in-hospital mortality prediction lack interpretability.

**Objective:**

This study aims to establish an explainable deep learning model to provide individualized in-hospital mortality prediction and risk factor assessment for patients with AMI.

**Methods:**

In this retrospective multicenter study, we used data for consecutive patients hospitalized with AMI from the Chongqing University Central Hospital between July 2016 and December 2022 and the Electronic Intensive Care Unit Collaborative Research Database. These patients were randomly divided into training (7668/10,955, 70%) and internal test (3287/10,955, 30%) data sets. In addition, data of patients with AMI from the Medical Information Mart for Intensive Care database were used for external validation. Deep learning models were used to predict in-hospital mortality in patients with AMI, and they were compared with linear and tree-based models. The Shapley Additive Explanations method was used to explain the model with the highest area under the receiver operating characteristic curve in both the internal test and external validation data sets to quantify and visualize the features that drive predictions.

**Results:**

A total of 10,955 patients with AMI who were admitted to Chongqing University Central Hospital or included in the Electronic Intensive Care Unit Collaborative Research Database were randomly divided into a training data set of 7668 (70%) patients and an internal test data set of 3287 (30%) patients. A total of 9355 patients from the Medical Information Mart for Intensive Care database were included for independent external validation. In-hospital mortality occurred in 8.74% (670/7668), 8.73% (287/3287), and 9.12% (853/9355) of the patients in the training, internal test, and external validation cohorts, respectively. The Self-Attention and Intersample Attention Transformer model performed best in both the internal test data set and the external validation data set among the 9 prediction models, with the highest area under the receiver operating characteristic curve of 0.86 (95% CI 0.84-0.88) and 0.85 (95% CI 0.84-0.87), respectively. Older age, high heart rate, and low body temperature were the 3 most important predictors of increased mortality, according to the explanations of the Self-Attention and Intersample Attention Transformer model.

**Conclusions:**

The explainable deep learning model that we developed could provide estimates of mortality and visual contribution of the features to the prediction for a patient with AMI. The explanations suggested that older age, unstable vital signs, and metabolic disorders may increase the risk of mortality in patients with AMI.

## Introduction

### Background

Acute myocardial infarction (AMI) refers to myocardial ischemia caused by acute blockage of coronary arteries, which is one of the most common cardiovascular emergencies [1]. AMI remains a leading cause of mortality worldwide, and patients with AMI have an estimated in-hospital mortality of 7% to 14% [2,3]. Therefore, the prediction of in-hospital mortality and assessment of risk factors for patients with AMI is of great interest to clinicians. Predicting the risk of mortality and conducting an individualized analysis of the risk factors in patients with AMI at initial diagnosis is critical for developing targeted interventions and improving the prognosis [4].

A substantial effort has been devoted to developing models for predicting mortality in patients with AMI [5]. In the past, generalized linear models (eg, the logistic regression [LR] model and Cox proportional hazard model) have been used to predict mortality in patients with AMI [6-9]. However, the generalized linear models fail to capture the nonlinear relations of the massive, high-dimensional, and incomplete medical data, which hinder the practical use and further clinical application of the models [10]. With the rapid development of artificial intelligence, tree-based machine learning models, such as random forest (RF) [11] and extreme gradient boosting (XGBoost) [12], were used to address problems related to the prediction of mortality and achieved promising performance [13-15]. Nevertheless, tree-based models tend to be ineffective in the face of web-based learning and sparse classification features. More importantly, tree-based models failed to develop the model with powerful deep learning modules through the backpropagation method, which limits the further improvement of the model performance [16]. Deep learning is the most advanced branch of artificial intelligence, which can automatically learn feature representations from training data and provide new prospects for solving the existing limitations in tree-based models [17]. However, state-of-the-art deep learning models specific to tabular data have not been applied to the prediction of mortality in patients with AMI. In addition, while a complex model may perform better than a simpler one, it could lead to black-box problems, meaning that only the inputs and outputs of the model can be seen and it is difficult to understand how the inputs affect the predictions, which limit their clinical acceptance and raise ethical and legal questions [18]. The proposal of methods that could provide explanations for black-box models might facilitate clinicians in understanding predictions and making faster and more accurate treatment decisions [9,19,20]. At present, there is a lack of tools to predict in-hospital mortality of patients with AMI based on tabular data–specific deep learning models, and the potential of state-of-the-art deep learning models for predicting mortality in AMI is unclear. In addition, most mortality prediction models for patients with AMI proposed in previous studies are black-box models, which makes it difficult for clinicians to understand the predictions and use the models in clinical practice.

### Contribution

In this study, we chose 3 state-of-the-art deep learning models to predict in-hospital mortality of patients with AMI and compared their performance with linear and tree-based models. In addition, we used an algorithm to interpret the best-performing black-box prediction model to obtain a tool for individualized mortality prediction and risk factor assessment in patients with AMI.

## Methods

### Data Source

This retrospective study included consecutive patients hospitalized with AMI from Chongqing University Central Hospital between July 2016 and December 2022, the Medical Information Mart for Intensive Care-IV (MIMIC-IV; version 2.2) database [21], and the Electronic Intensive Care Unit Collaborative Research Database (eICU-CRD; version 2.0) [22]. Chongqing University Central Hospital is a city-designated emergency tertiary medical center in China with a permanent population of >32 million. The MIMIC-IV database and eICU-CRD are 2 large free public databases. The MIMIC-IV database contains case information from 299,712 patients admitted to the intensive care unit or the emergency department at the Beth Israel Deaconess Medical Center from 2008 to 2019. The eICU-CRD included information from 139,367 patients from 208 hospitals in the United States between 2014 and 2015.

### Ethical Considerations

Access to the MIMIC-IV database and eICU-CRD was approved by the Massachusetts Institute of Technology (Cambridge, Massachusetts) and Beth Israel Deaconess Medical Center (Boston, Massachusetts), and consent was obtained for the data collection. These databases are public, all patient data are anonymized, and data extracted from the databases do not require individual informed consent. PX attended a series of courses offered by the National Institutes of Health and was granted access to these databases after passing the required assessment (record ID 51524821). The study was conducted following the Declaration of Helsinki. Patient data obtained from the Chongqing University Central Hospital were also anonymized. Ethics approval was obtained from the Chongqing University Central Hospital Ethics Committee, and informed consent was waived.

### Population

**Figure 1:**
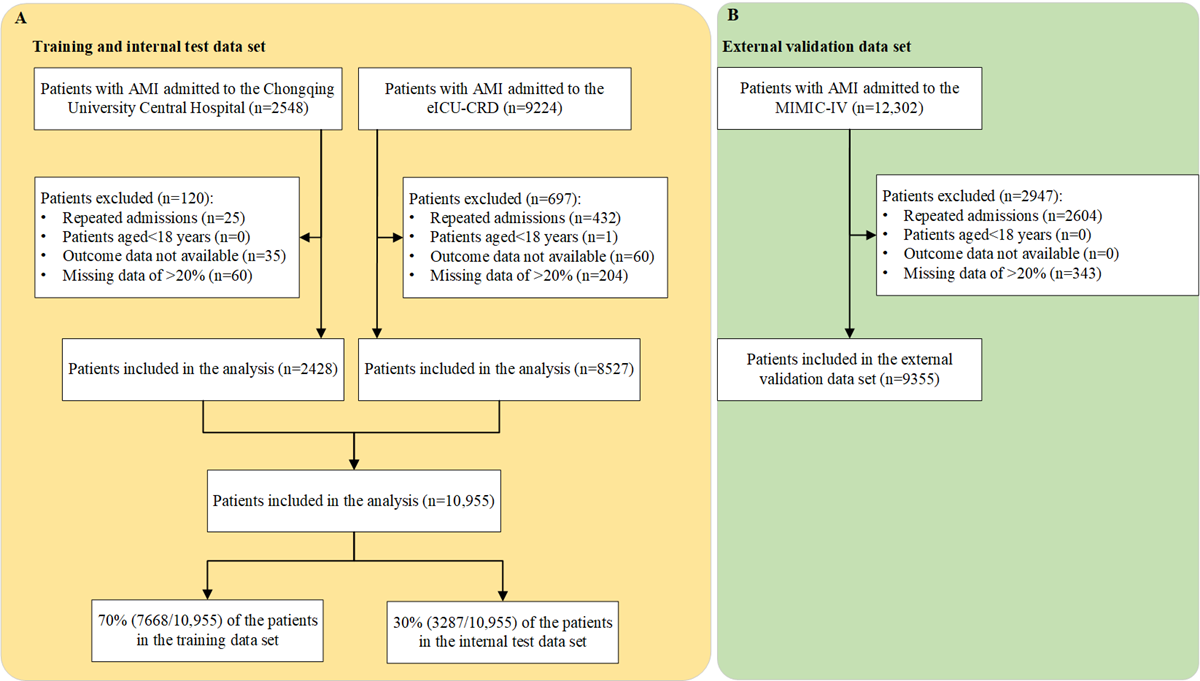
Study profile: (A) Inclusion and exclusion criteria for the training and internal test data set; and (B) Inclusion and exclusion criteria for the external validation data set. AMI: acute myocardial infarction; eICU-CRD: Electronic Intensive Care Unit Collaborative Research Database; MIMIC-IV: Medical Information Mart for Intensive Care-IV.

All patients diagnosed with AMI according to the *ICD* (*International Classification of Diseases*) in the Chongqing University Central Hospital between July 2016 and December 2022, MIMIC-IV database and eICU-CRD were included. *ICD-9* (*International Classification of Diseases, Ninth Revision*) code 410 and *ICD-10* (*International Statistical Classification of Diseases, Tenth Revision*) code I21 were used to identify the patients with AMI. For patients with AMI admitted multiple times during the study period, only the first admission was included. Exclusion criteria were (1) patients aged <18 years, (2) patients with unclear clinical outcome, and (3) patients with >20% of missing data (Figure 1).

### Data Extraction

Patient data at Chongqing University Central Hospital were collected by reviewing electronic medical records. The data in the MIMIC-IV database and eICU-CRD were obtained using SQL. A total of 39 variables were incorporated into this study to determine the prediction models, including demographics, vital signs, laboratory parameters, comorbidities and drug use data based on previous studies, and clinical relevance and experience. All vital signs and laboratory data were the first measurements after admission. Only variables with <30% of missing values across the entire data set were considered for inclusion. This inclusion criterion, unlike the exclusion criterion for patients, was applicable to variables in the entire database. The predetermined threshold was selected according to the evidence from previous studies and experience [23,24]. The missing values were filled by the K-nearest neighbors (KNN) algorithm, and the hyperparameter K for KNN was determined using a 5-fold cross-validation in the training data set. The KNN model trained in the training data set was then used to fill in the missing values in the internal test and external validation data sets. In total, 24,004 missing data points were successfully filled by the KNN model. The clinical outcome assessed in this study was all-cause in-hospital mortality.

### Statistical Analysis

Patients with AMI in the training data set were divided into a survival group and a nonsurvival group according to the clinical outcome. Shapiro-Wilk test was applied for the normality test. Continuous variables were presented as medians (IQRs), and categorical variables were presented as numbers and percentages. To test the differences within the groups, the 2-tailed *t* test and Kruskal-Wallis test were used for normal and nonnormal continuous variables, respectively. For categorical variables, between-group differences were evaluated using the chi-square test. *P* value <.05 was regarded as statistically significant.

### Model Development

The data of the Chongqing University Central Hospital and eICU-CRD were merged and shuffled. The shuffled data set was then randomly divided into the training data set and internal test data set in a 7:3 ratio. The reason for this combination was that the data of the eICU-CRD were collected from 208 hospitals in the United States, and the generalization ability of the model may be improved by combining the data of eICU-CRD with the data from Chongqing University Central Hospital into a larger multicenter training data set. Patient data in the MIMIC-IV database were used for an independent external validation, which is independent of the Chongqing University Central Hospital and eICU-CRD data.

Data preprocessing tasks were performed before training the prediction models. The KNN algorithm and *z* score were used to fill in missing values and normalize continuous variables, respectively.

A total of 9 models were used to predict in-hospital mortality in patients with AMI, including state-of-the-art deep learning models (eg, Self-Attention and Intersample Attention Transformer [SAINT] [25], TabTransformer [26], and TabNet [27]), a basic deep learning model (eg, multilayer perceptron [MLP] [28]), tree-based machine learning models (eg, RF [11], XGBoost [12], light gradient boosting machine [29], and categorical boosting [30]), and a linear model (LR [31]). An optimization procedure based on nested 5-fold cross-validation Bayesian optimization was used for each model with 100 iterations to determine the hyperparameters. Additional details on hyperparameters setting are provided in Tables S1-S8 in Multimedia Appendix 1. The complete training data set was then used to train the final models with the optimal hyperparameters. Code for model development is publicly available [32].

### Model Evaluation

The area under the receiver operating characteristic curve (AUC) was used to assess the performance of the prediction models in the internal test data set and external validation data set. The cutoff thresholds were determined according to the Youden index, and 5 metrics were further calculated, including balanced accuracy, sensitivity, specificity, precision, and *F*_1_-score. CIs were calculated using 1000 bootstrap samples. The calibration curve and Brier score were used to evaluate the reliability of probability estimates of the prediction models; decision curve analysis was used to evaluate the clinical utility of the models.

### Model Explanation

The Shapley Additive Explanations (SHAP) algorithm was used to interpret the predictions to obtain global and local influences of the relevant features on the predictions. With a set of feature values of a patient, a SHAP value represents how much a single feature’s value influences the difference between the actual prediction and the average prediction of the model in interaction with other features. Briefly, the mean prediction of the model plus the sum of the SHAP values of a sample is equal to the prediction of that sample [33,34].

We first used the SHAP algorithm to visualize the global relative importance of features to the predictions. The rationality of the abovementioned results was then verified by statistical methods. Patients in the training data set were further divided into a survival group and a nonsurvival group. Statistical test was used to evaluate the differences in features between the groups, and the test results were compared with those obtained by the SHAP algorithm. Finally, we linearly mapped the SHAP value to the probability of driving an increase or decrease in predicted mortality, quantifying and visualizing the contribution of each feature to the prediction in individuals.

## Results

### Statistical Analysis Results

A total of 2428 consecutive patients with AMI who were admitted to Chongqing University Central Hospital between July 2016 and December 2022 and 8527 patients with AMI from the eICU-CRD were included in the analysis. A total of 10,955 patients were randomly divided into a training data set of 7668 (70%) patients and an internal test data set of 3287 (30%) patients. The median age of patients in the training and internal test data sets was 66 (IQR 56-76) years, with 65.6% (5030/7668) and 68.09% (2238/3287) of male participants, respectively. In total, 8.74% (670/7668) of the patients each in the training data set and 8.73% (287/3287) of the patients in the internal test data set died during their hospital stay (Table 1).

A total of 9355 patients with AMI from the MIMIC-IV database were included for independent external validation. The median age of patients in the external validation group was 72 (IQR 62-82) years, higher than that in the training or internal test data set. In total, 61.17% (5722/9355) of the patients were male participants, and 9.12% (853/9355) of the patients died during the hospitalization (Table 1).

**Table 1:** Baseline characteristics of patients with acute myocardial infarction included in the models (N=10,955).

Variables		Training data set (n=7668)	Internal test data set (n=3287)	External validation data set (n=9355)
**Demographic data**				
	Age (years), median (IQR)	66 (56-76)	66 (56-76)	72 (62-82)
	Female participants, n (%)	2638 (34.4)	1049 (31.9)	3633 (38.8)
**Vital signs,** **median (IQR)**				
	Heart rate (beats/minute)	81 (70-96)	81 (70-95)	83 (72-96)
	Respiratory rate (minute^−1^)	20 (17-21)	20 (17-21)	18 (16-20)
	Systolic blood pressure (mm Hg)	129 (111-149)	129 (112-148)	126 (109-145)
	Diastolic blood pressure (mm Hg)	76 (64-88)	76 (64-88)	69 (58-82)
	Temperature (°C)	36.6 (36.4-36.8)	36.6 (36.4-36.8)	36.7 (36.4-37)
**Laboratory data, median (IQR)**				
	Troponin T or I (ng/mL)	0.74 (0.10-4.85)	0.69 (0.10-4.93)	0.32 (0.11-1.01)
	White blood cell (k/µ)	10.6 (8.2-13.6)	10.7 (8.3-13.7)	9.30 (7.1-12.4)
	Red blood cell (m/µ)	4.42 (3.89-4.87)	4.43 (3.91-4.87)	3.88 (3.34-4.41)
	Hemoglobin (g/dL)	13.5 (11.7-14.8)	13.4 (11.8-14.9)	11.6 (9.9-13.2)
	Hematocrit (%)	40.1 (35.4-43.9)	40.2 (35.7-44)	35.2 (30.5-39.6)
	Platelet (k/µ)	215 (173-265)	217 (174-268)	208 (164-262)
	Prothrombin time (seconds)	13.3 (12.3-14.5)	13.3 (12.3-14.5)	12.9 (11.8-14.7)
	International normalized ratio	1.06 (1-1.2)	1.05 (1-1.2)	1.2 (1.1-1.3)
	Sodium (mEq/L)	138 (136-140)	138 (136-140)	139 (137-141)
	Potassium (mEq/L)	4.00 (3.70-4.37)	4 (3.7-4.4)	4.2 (3.8-4.5)
	Chloride (mEq/L)	103.3 (100-106)	103.4 (100-106)	103 (100-106)
	Calcium (mg/dL)	8.90 (8.5-9.3)	8.9 (8.41-9.3)	8.7 (8.3-9.1)
	Magnesium (mg/dL)	1.99 (1.8-2.2)	1.99 (1.77-2.19)	2 (1.8-2.2)
	Creatinine (mg/dL)	1.04 (0.84-1.38)	1.03 (0.82-1.40)	1.1 (0.8-1.6)
	Blood urea nitrogen (mg/dL)	19 (14- 28)	19 (14-28)	21 (15-35)
**Comorbidities, n (%)**				
	Hypertension	4616 (60.2)	1965 (59.8)	3962 (42.4)
	Hyperlipidemia	820 (10.7)	397 (12.1)	4940 (52.8)
	Diabetes	2516 (32.8)	1087 (33.1)	3813 (40.8)
	Heart failure	1987 (25.9)	854 (26)	4298 (45.9)
	Renal failure	1648 (21.5)	760 (23.1)	4446 (47.5)
	Cerebral vascular disease	788 (10.3)	348 (10.6)	1106 (11.8)
	Peripheral vascular disease	1062 (13.8)	439 (13.4)	1324 (14.2)
	Chronic obstructive pulmonary disease	864 (11.3)	386 (11.7)	688 (7.4)
**Operations, n (%)**				
	Coronary arteriography	3855 (50.3)	1663 (50.6)	2839 (30)
	Percutaneous coronary intervention	3083 (40.2)	1359 (41.3)	2954 (31.6)
**Drugs, n (%)**				
	Aspirin	4941 (64.4)	2126 (64.7)	8534 (91.2)
	Clopidogrel	2070 (27)	872 (26.5)	4385 (46.9)
	Ticagrelor	1491 (19.4)	629 (19.1)	622 (6.6)
	Angiotensin-converting enzyme inhibitor	1827 (23.8)	765 (23.3)	4001 (42.8)
	Angiotensin receptor blockers	240 (3.1)	118 (3.6)	1158 (12.4)
	Statins	3728 (48.6)	1596 (48.6)	8276 (88.5)
	β-Blockers	3565 (46.5)	1555 (47.3)	7625 (81.5)
**Clinical outcome**				
	All-cause in-hospital mortality, n (%)	670 (8.7)	287 (8.7)	853 (9.1)
	Length of hospital stay (days), median (IQR)	4.98 (2.50-9.25)	4.95 (2.42-9.51)	5.33 (2.80-9.85)

### Model Performance

**Figure 2:**
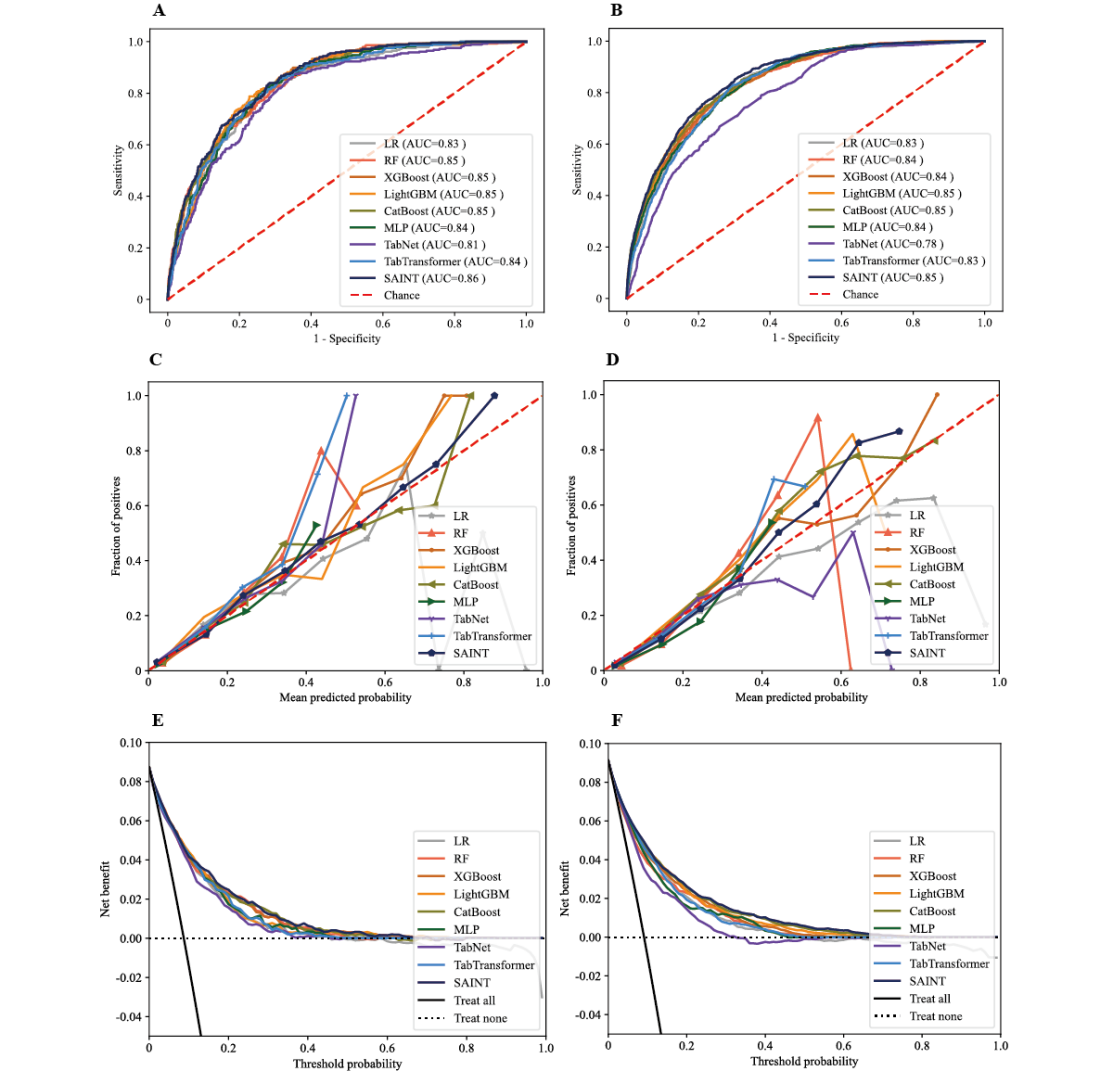
Performance of the models in predicting in-hospital mortality of patients with acute myocardial infarction in the internal test set and external validation set: (A) and (B), AUCs of the models; (C) and (D), calibration plots of the models; and (E) and (F), decision curves of the models. AUC: area under the receiver operating characteristic curve; CatBoost: categorical boosting; LightGBM: light gradient boosting machine; LR: logistic regression; MLP: multilayer perceptron; RF: random forest; SAINT: Self-Attention and Intersample Attention Transformer; XGBoost: extreme gradient boosting.

The AUCs of 9 models in the internal test data set and external validation data set are shown in Figures 2A and 2B (AUC=1 indicates perfect prediction and AUC=0.5 indicates random prediction), and the other 5 evaluation metrics, including balanced accuracy, sensitivity, specificity, precision, and *F*_1_-score are listed in Tables 2 and 3.

**Table 2:** Values of the evaluation metrics of the models in the internal test data set.

Models	Balanced accuracy (95% CI)	Sensitivity (95% CI)	Specificity (95% CI)	Precision (95% CI)	*F*_1_-score (95% CI)	AUC^a^ (95% CI)
Logistic regression	0.76 (0.74-0.78)	0.85 (0.81-0.89)	0.67 (0.65-0.68)	0.20 (0.17-0.22)	0.32 (0.29-0.35)	0.83 (0.81-0.85)
Random forest	0.72 (0.69-0.75)	0.58 (0.52-0.64)	0.87 (0.86-0.88)	0.30 (0.26-0.34)	0.39 (0.35-0.43)	0.85 (0.82-0.87)
Extreme gradient boosting	0.75 (0.73-0.78)	0.74 (0.69-0.78)	0.77 (0.75-0.78)	0.23 (0.21-0.26)	0.35 (0.32-0.39)	0.85 (0.83-0.87)
Light gradient boosting machine	0.71 (0.68-0.73)	0.52 (0.47-0.58)	0.89 (0.88-0.91)	0.32 (0.28-0.36)	0.40 (0.35-0.44)	0.85 (0.83-0.87)
Categorical boosting	0.76 (0.73-0.78)	0.74 (0.69-0.79)	0.78 (0.76-0.80)	0.24 (0.21-0.27)	0.37 (0.33-0.40)	0.85 (0.83-0.87)
Multilayer perceptron	0.77 (0.74-0.79)	0.79 (0.75-0.84)	0.74 (0.73-0.76)	0.23 (0.20-0.25)	0.32 (0.35-0.39)	0.84 (0.82-0.86)
TabNet	0.76 (0.73-0.78)	0.86 (0.82-0.90)	0.65 (0.64-0.67)	0.19 (0.17-0.21)	0.31 (0.28-0.34)	0.81 (0.79-0.84)
TabTransformer	0.77 (0.75-0.79)	0.85 (0.81-0.89)	0.69 (0.67-0.71)	0.21 (0.18-0.23)	0.33 (0.30-0.37)	0.84 (0.82-0.86)
Self-Attention and Intersample Attention Transformer	0.76 (0.73-0.78)	0.74 (0.69-0.79)	0.77 (0.75-0.78)	0.23 (0.21-0.26)	0.36 (0.32-0.39)	0.86 (0.84-0.88)

^a^AUC: area under the receiver operating characteristic curve.

**Table 3:** Values of the evaluation metrics of the models in the external validation data set.

Models	Balanced accuracy (95% CI)	Sensitivity (95% CI)	Specificity (95% CI)	Precision (95% CI)	*F*_1_-score (95% CI)	AUC^a^ (95% CI)
Logistic regression	0.73 (0.74-0.76)	0.91 (0.89-0.93)	0.58 (0.57-0.59)	0.18 (0.17-0.19)	0.30 (0.28-0.31)	0.83 (0.82-0.85)
Random forest	0.75 (0.73-0.77)	0.70 (0.67-0.73)	0.80 (0.79-0.81)	0.26 (0.24-0.28)	0.38 (0.36-0.40)	0.84 (0.82-0.85)
Extreme gradient boosting	0.75 (0.74-0.77)	0.82 (0.79-0.84)	0.69 (0.68-0.70)	0.21 (0.19-0.22)	0.33 (0.31-0.35)	0.84 (0.83-0.85)
Light gradient boosting machine	0.73 (0.71-0.75)	0.57 (0.54-0.61)	0.89 (0.88-0.89)	0.34 (0.31-0.36)	0.42 (0.40-0.45)	0.85 (0.85-0.86)
Categorical boosting	0.76 (0.75-0.77)	0.81 (0.78-0.84)	0.71 (0.70-0.72)	0.22 (0.21-0.23)	0.35 (0.33-0.36)	0.85 (0.83-0.86)
Multilayer perceptron	0.75 (0.74-0.76)	0.89 (0.86-0.91)	0.62 (0.61-0.63)	0.19 (0.18-0.20)	0.31 (0.29-0.33)	0.84 (0.82-0.85)
TabNet	0.69 (0.68-0.70)	0.93 (0.91-0.94)	0.45 (0.44-0.46)	0.14 (0.14-0.15)	0.25 (0.24-0.26)	0.78 (0.77-0.80)
TabTransformer	0.75 (0.74-0.76)	0.92 (0.90-0.94)	0.57 (0.57-0.59)	0.18 (0.17-0.19)	0.30 (0.28-0.31)	0.83 (0.82-0.85)
Self-Attention and Intersample Attention Transformer	0.77 (0.76-0.78)	0.87 (0.85-0.90)	0.67 (0.66-0.68)	0.21 (0.20-0.22)	0.34 (0.32-0.36)	0.85 (0.84-0.87)

^a^AUC: area under the receiver operating characteristic curve.

In general, the SAINT model performed best in both the internal test data set and the external validation data set among the 9 prediction models, with an AUC of 0.86 (95% CI 0.84-0.88) and 0.85 (95% CI 0.84-0.87), respectively. The corresponding balanced accuracy of the internal test data set and external validation data set were 0.76 (95% CI 0.73-0.78) and 0.77 (95% CI 0.76-0.78), sensitivities were 0.74 (95% CI 0.69-0.79) and 0.87 (95% CI 0.85-0.90), specificities were 0.77 (95% CI 0.75-0.78) and 0.67 (95% CI 0.66-0.68), precision values were 0.23 (95% CI 0.21-0.26) and 0.21 (95% CI 0.20-0.22), and *F*_1_-score were 0.36 (95% CI 0.32-0.39) and 0.34 (95% CI 0.32-0.36), respectively. The calibration curves demonstrated good consistency between the predicted and observed probabilities for the SAINT model with curves close to the 45° line, and the Brier scores were 0.07 (95% CI 0.01-0.21) and 0.07 (95% CI 0.02-0.17) in the internal test data set and external validation data set, respectively (Figures 2C and 2D). In addition, the decision curve analysis (Figures 2E and 2F) illustrated that the SAINT model was clinically beneficial in both the internal test data set and external validation data set.

Moreover, we tried to train the model using different data sets and evaluated the performance of the models in different external validation data sets. The results showed that the SAINT model performed better than other models (Figures S2 and S3 and Tables S9-S12 in Multimedia Appendix 1). Furthermore, we evaluated the model’s predictive power using the measurements obtained within the first 2 hours of patient admission. This evaluation aimed to assess the predictive power of the models for the early diagnostic stage of patients with AMI. The results demonstrated that although the SAINT model still outperformed the other models, there was a clear decrease in the predictive power of all models, with an average decrease of 0.06 (SD 0.02) in the AUC. (Figure S4 and Table S13 in Multimedia Appendix 1).

### Model Explanation Results

**Figure 3:**
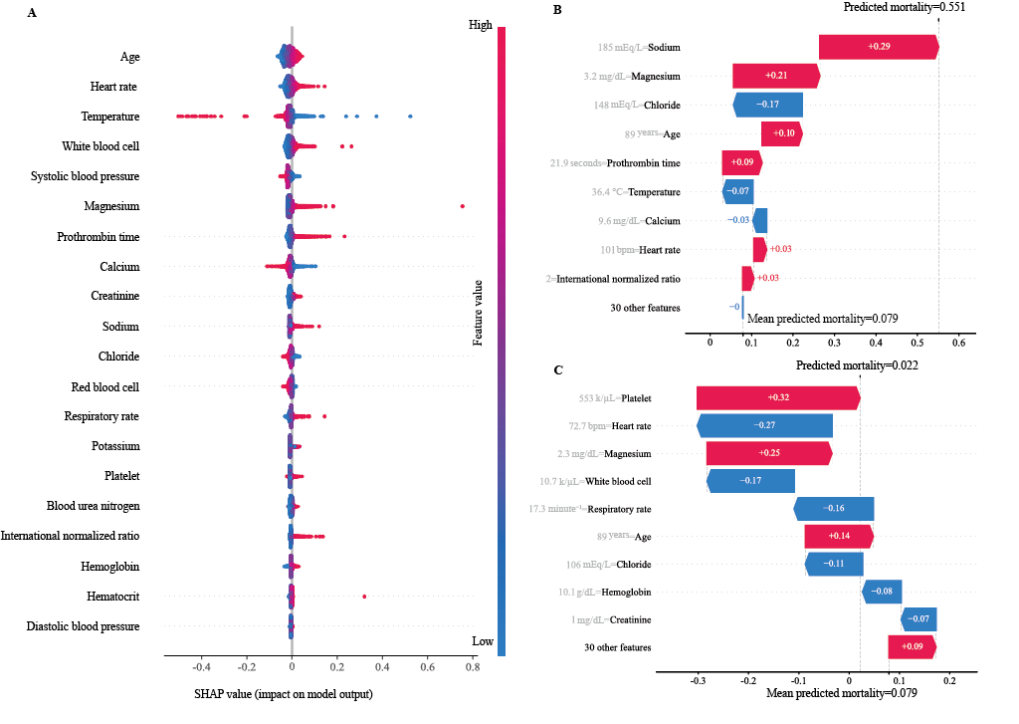
The impact of the input features on predictions: (A) the effect of the input features on the model at the global level. Each dot represents the influence of a feature on the prediction for a patient. Red dots indicate a higher value of the features, and blue dots indicate a lower value of the features. A dot on the left side of the x-axis represents that the feature value decreased mortality prediction, and a dot on the right side of the x-axis represents that the feature value increased mortality prediction, (B) an example of risk factor analysis for a patient in the external validation set (in-hospital death), and (C) an example of risk factor analysis for a patient in the external validation set (survival at hospital discharge). SHAP: Shapley Additive Explanations.

The relative importance and contribution of the top 20 features to the SAINT model are shown in Figure 3A, which was obtained by the SHAP algorithm interpreting the predictions of the SAINT model. The importance and contribution of the full features to the SAINT model are presented in Figure S1 in Multimedia Appendix 1. Age was ranked as the most important feature for the prediction model, followed by heart rate, temperature, white blood cell level, systolic blood pressure, magnesium level, prothrombin time, calcium level, creatinine level, and sodium level. Considering the impact of age on the predictions as an example, advanced age was commonly associated with a higher risk of mortality, and younger age generally drove the predictions toward survival (red dots represent a high value of the features, which on the right side of the x-axis represents an increased risk of mortality). Similar explanations can be applied to other features, and most interpretations were consistent with clinical experience. It is notable that features can drive predictions in different directions (increasing or decreasing mortality predictions) for patients with different features, unlike previous scoring systems (eg, Global Registry of Acute Coronary Events risk score [35] and Thrombolysis in Myocardial Infarction score [36]), where the influence of a given feature value on the prediction is invariant. The 9 most important features for the prediction models showed a statistically significant difference between the survival group and the nonsurvival group in the training data set (*P*<.05), which further supports the rationality of the explanations of the features (Table 4).

We further mapped the SHAP values to the probability of driving an increase or decrease in mortality and visualized the application of the method to 1 deceased patient and 1 surviving patient in the external validation data set (Figures 3B and 3C). In the case of the deceased patient, the patient was female and aged 89 years with a history of heart failure and renal failure. The model predicted that the risk of mortality of the patients was 0.551. High levels of serum sodium (185 mEq/L) and serum magnesium (3.2 mg/dL) drove a 0.29 and 0.21 increase in the risk of mortality, respectively, whereas a high serum chloride level was associated with a 0.17 reduction in the risk of mortality. A similar explanation can be applied to other features. The prediction was driven by 39 features used for model training, and the sum of the SHAP values for all features plus the baseline risk equaled the predicted risk of mortality. The baseline risk score was obtained by calculating the average predicted risk of mortality among all patients in the training data set (0.079). Therefore, the SHAP algorithm made our model explainable at both the global and individual levels.

**Table 4:** Baseline characteristics of patients with acute myocardial infarction in the training data set by clinical outcomes (n=7668).

Variables				Survival group (n=6998)		Nonsurvival group (n=670)		*P* value^a^
**Demographic data**								
	Age (years), median (IQR)		66 (56-75)		75 (66-83)		*<.001*	
	**Sex, n (%)**							<.001
		Male	4661 (66.6)		369 (55.1)			
		Female	2337 (33.4)		301 (44.9)			
**Vital signs, median (IQR)**								
	Heart rate (beats/minute)		80 (69-94)		92 (78-108)		*<.001*	
	Respiratory rate (minute^−1^)		20 (17-21)		20 (18-25)		*<.001*	
	Systolic blood pressure (mm Hg)		130 (113-149)		116 (98-142)		*<.001*	
	Diastolic blood pressure (mm Hg)		77 (65-88)		68 (57-81)		*<.001*	
	Temperature (°C)		36.6 (36.4-36.8)		36.5 (36.1-36.8)		*<.001*	
**Laboratory data, median (IQR)**								
	Troponin T or I (ng/mL)		0.66 (0.10-4.47)		1.77 (0.26-8.78)		*<.001*	
	White blood cell (k/µ)		10.4 (8.2-13.4)		12.4 (9.1-16.8)		*<.001*	
	Red blood cell (m/µ)		4.45 (3.94-4.88)		4.05 (3.48-4.59)		*<.001*	
	Hemoglobin (g/dL)		13.6 (11.8-14.9)		12.1 (10.3-14)		*<.001*	
	Hematocrit (%)		40.4 (35.9-44)		37 (32.1-42.4)		*<.001*	
	Platelet (k/µ)		215 (174-264)		213 (153-270)		.06	
	Prothrombin time (seconds)		13.2 (12.2-14.3)		14.5 (13.2-17.2)		*<.001*	
	International normalized ratio		1.04 (1-1.16)		1.2 (1.08-1.49)		*<.001*	
	Sodium (mEq/L)		138 (136-140)		138 (135-141)		.06	
	Potassium (mEq/L)		4 (3.7-4.3)		4.2 (3.73-4.70)		*<.001*	
	Chloride (mEq/L)		103.6 (100.4-106)		103 (98.5-106)		*<.001*	
	Calcium (mg/dL)		8.9 (8.5-9.3)		8.6 (8.18-9.10)		*<.001*	
	Magnesium (mg/dL)		1.99 (1.80-2.19)		2 (1.71-2.30)		*<.001*	
	Creatinine (mg/dL)		1.01 (0.82-1.30)		1.42 (1.06-2.14)		*<.001*	
	Blood urea nitrogen (mg/dL)		19 (14-27)		27 (18-41)		*<.001*	
**Comorbidities, n (%)**								
	Hypertension		4209 (60.1)		407 (60.7)		.76	
	Hyperlipidemia		767 (11)		53 (7.9)		*.02*	
	Diabetes		2249 (32.1)		267 (39.9)		*<.001*	
	Heart failure		1688 (24.1)		299 (44.6)		*<.001*	
	Renal failure		1333 (19)		315 (47)		*<.001*	
	Cerebral vascular disease		670 (9.6)		118 (17.6)		*<.001*	
	Peripheral vascular disease		971 (13.9)		91 (13.6)		.83	
	Chronic obstructive pulmonary disease		749 (10.7)		115 (17.2)		*<.001*	
**Operations, n (%)**								
	Coronary arteriography		3670 (52.4)		185 (27.6)		*<.001*	
	Percutaneous coronary intervention		2920 (41.7)		163 (24.3)		*<.001*	
**Drugs, n (%)**								
	Aspirin		4583 (65.5)		358 (53.4)		*<.001*	
	Clopidogrel		1943 (27.8)		127 (19)		*<.001*	
	Ticagrelor		1413 (20.2)		78 (11.6)		*<.001*	
	Angiotensin-converting enzyme inhibitor		1777 (25.4)		50 (7.5)		*<.001*	
	Angiotensin receptor blocker		233 (3.3)		7 (1)		*.001*	
	Statins		3512 (50.2)		216 (32.2)		*<.001*	
	β-Blockers		3369 (48.1)		196 (29.3)		*<.001*	
**Clinical outcome, median (IQR)**								
	Length of hospital stay (days)		5.15 (2.59-9.34)		3.71 (1.23-8.27)		*<.001*	

^a^Italicized *P* values (<.05) were considered statistically significant.

## Discussion

### Principal Findings

In this multicenter, retrospective study, we trained 3 state-of-the-art deep learning models on patients with AMI from Chongqing University Central Hospital and patient data from the eICU-CRD and compared their performance with 4 advanced tree-based models, an LR model, and an MLP. We performed both internal and external validation (MIMIC-IV database) to confirm the predictive ability of the models and ensure the reliability of the results. In addition, the SHAP algorithm was used to interpret the predictions of the best-performing model (SAINT) to quantify and visualize the impact of the features on the predicted mortality at global and individual levels.

The models developed in this study had similar AUC in the internal test data set and the external validation data set, which demonstrated a good generalization ability of the models. The training data set we used was collected from 209 centers in Asia and the United States, and the multicenter data contributed to improving the performance of the model, especially in terms of generalization ability. However, although the specificities of the models in the internal test data set were generally higher and the sensitivities were lower than that in the external validation, the models tended to overestimate patient mortality in the external validation data set, leading to false positive results. The SAINT model is a hybrid deep learning–based approach specifically designed to solve tabular data problems and outperforms tree-based models over a variety of benchmark tasks [25]. In this study, the SAINT model performed best in both the internal test data set and the external validation data set among the 9 prediction models, with the highest AUC and lowest Brier score. In addition, the decision curve analysis indicated that the SAINT model could provide net benefits across an extensive range of threshold values. The SAINT model attained the highest balanced accuracy during evaluation on the external validation data set, indicating that the model had strong classification ability. Nevertheless, it is worth noting that both precision and specificity metrics associated with the SAINT model did not exhibit high values, implying a proclivity of the model to overestimate the mortality risk among patients with AMI. According to these results, state-of-the-art deep learning methods have greater potential in accurately predicting mortality in patients with AMI than linear, MLP, and tree-based models.

The SHAP algorithm was used to obtain an interpretation of the predictions of the SAINT model, and the impact of most features on the predictions is consistent with clinical practice and previous evidence. For instance, advanced age, unstable vital signs, presence of infection, metabolic disorders, and coagulation disorders lead to increased predicted mortality. The results of the model interpretation indicated that advanced age was ranked as the most important risk factor for mortality. In addition, as patients age, their physical function and resistance decline, which may increase their risk of mortality [37,38]. Unstable vital signs (eg, elevated heart rate and respiratory rate and decreased levels of systolic blood pressure and temperature) usually indicate that the patient is in a critical condition, and such patients tend to have a worse prognosis [39,40]. Similarly, the presence of infection (eg, elevated levels of white blood cells) may impair physical function and reduce the effectiveness of treatment [41]. In addition, metabolic and coagulation disorders (eg, elevated levels of magnesium and sodium and increased prothrombin time) are associated with an increased risk of mortality [42].

LR models and Cox proportional hazard models are commonly used tools for predicting the mortality of patients with AMI [6]. However, the rapid development of medical information technology brings massive, nonlinear, and high-dimensional data, which challenge the generalized linear model [43]. Tree-based machine learning models (eg, RF, XGBoost, and light gradient boosting machine) achieved substantial gains in the predictive accuracy of tabular data [4,13,14]. Nevertheless, tree-based models were developed directly using the information entropy of data without calculating the loss function and performing backpropagation, which limits the model from taking advantage of powerful deep learning modules and conducting web-based learning [29,30]. Recently, deep learning models have received extensive attention in the screening, diagnosis, and prognosis prediction of diseases, and their performance even outperformed that of clinicians in some aspects [44]. A recent, large population-based study by Mohammad et al [45] revealed the tremendous potential of deep learning models to identify the risk of adverse outcomes in patients with AMI. However, the prediction model used in this study is the most basic deep learning model (MLP), and the ability of state-of-the-art deep learning models to predict the mortality of patients with AMI remains unclear. In addition, the model used in the study by Mohammad et al [45] is regarded as a black-box model due to the complex internal calculation process, that is, we can only see the input and final predictions of the model but cannot understand how the model makes decisions. We used the SHAP algorithm to quantify and visualize the features that drive the predictions, which combines the advantages of complex deep learning models and simple linear models to solve the problem of insufficient predictive ability of the linear model and black-box model of the complex deep learning model. The effect of each feature on the prediction is not isolated but derived from the interaction with the other features, which makes it superior to the fixed feature weight in the linear models [33]. Furthermore, the SHAP algorithm not only provides the impact of the features on the model but also provides the interpretation at an individual level. Such a patient-level interpretable model allows clinicians to combine the predictions and explanations with their empirical knowledge to facilitate decision-making [19].

### Limitations

This study has several limitations. First, to enable the models to learn more comprehensive information and improve performance, up to 39 features were used to develop the model. Nevertheless, the variables included in the study were limited by the retrospective design and the use of publicly available databases. Second, there were missing values in the raw data; although the KNN algorithm was used to complete them, the real data could provide more valuable information. This limitation was particularly significant during the early stages of patient hospital admission when the available predictors were limited. Even with the application of the KNN algorithm to complete the missing values, the predictive power of the model could be compromised. Third, although the SHAP algorithm was used to obtain the explanations of the models, the impact of features on the predictions is not equal to the association in the causal chain. In addition, features such as age and sex, while influencing predictions, were not subject to human intervention. However, the explanations of the relationship between features and predictions may guide us in examining causation.

### Conclusions

In conclusion, in this multicenter study, we developed and externally validated 9 prediction models for in-hospital mortality in patients with AMI. Our results indicate that state-of-the-art deep learning models may provide a promising alternative solution to further improve the predictive ability of mortality in patients with AMI. The explanations of the model suggest that older age, unstable vital signs, and metabolic disorders may increase the risk of mortality in patients with AMI. Transparent and explainable models are expected to support clinical practice, trial design, and future research. However, future prospective studies are needed to confirm the findings.

## Appendix

Multimedia Appendix 1 Supplementary materials.

## Abbreviations

AMI: acute myocardial infarction
AUC: area under the receiver operating characteristic curve
eICU-CRD: Electronic Intensive Care Unit Collaborative Research Database
ICD: International Classification of Diseases
ICD-9: International Classification of Diseases, Ninth Revision
ICD-10: International Statistical Classification of Diseases, Tenth Revision
KNN: K-nearest neighbors
LR: logistic regression
MIMIC-IV: Medical Information Mart for Intensive Care-IV
MLP: multilayer perceptron
RF: random forest
SAINT: Self-Attention and Intersample Attention Transformer
SHAP: Shapley Additive Explanations
XGBoost: extreme gradient boosting

## Data Availability

The data sets from the Medical Information Mart for Intensive Care-IV database and Electronic Intensive Care Unit Collaborative Research Database presented in this study can be found in web-based repositories [46,47]. The data set from Chongqing University Central Hospital is not publicly available but is available from the corresponding author on reasonable request.
