# Supplementary material for: Development and Validation of an Explainable Deep Learning Model to Predict In-Hospital Mortality for Patients With Acute Myocardial Infarction: Algorithm Development and Validation Study
Source: J Med Internet Res. 2024 May 10;26:e49848. doi: 10.2196/49848 (PMC11127140; doi:10.2196/49848)
Supplement: Multimedia Appendix 1 [file jmir_v26i1e49848_app1.docx]

**Supplementary material**

**Supplemental Table 1:** **The hyperparameter optimization range and results of RF**

| Hyper-parameter | Tuning range | Tuning result |
| --- | --- | --- |
| max_depth | (2, 12) | 10 |
| n_estimators | (5, 100) | 70 |

RF, random forest.

**Supplemental Table 2: The hyperparameter optimization range and results of CatBoost**

| Hyper-parameter | Tuning range | Tuning result |
| --- | --- | --- |
| learning_rate | (0.01, 0.3) | 0.15 |
| max_depth | (2, 12) | 5 |
| l2_leaf_reg | (0.5, 30) | 4.34 |

CatBoost, Categorical Boosting.

**Supplemental Table 3:** **The hyperparameter optimization range and results of XGBoost**

| Hyper-parameter | Tuning range | Tuning result |
| --- | --- | --- |
| max_depth | (2, 12) | 4 |
| alpha | (1e-8, 1) | 0.06 |
| lamba | (1e-8, 1) | 2.63e-07 |
| eta | (0.01, 0.3) | 0.15 |

XGBoost, eXtreme Gradient Boosting.

**Supplemental Table 4:** **The hyperparameter optimization range and results of LightGBM**

| Hyper-parameter | Tuning range | Tuning result |
| --- | --- | --- |
| num_leaves | (2, 4096) | 538 |
| lamba_l1 | (1e-8, 10) | 5.83 |
| lamba_l2 | (1e-8, 10) | 2.71e-06 |
| learning_rate | (0.01, 0.3) | 0.09 |

LightGBM, Light Gradient Boosting Machine.

**Supplemental Table 5:** **The hyperparameter optimization range and results of MLP**

| Hyper-parameter | Tuning range | Tuning result |
| --- | --- | --- |
| hidden_dim | (10, 100) | 87 |
| n_layers | (2, 5) | 4 |
| learning_rate | (0.0005, 0.001) | 0.0008 |

MLP, multi-layer perceptron.

**Supplemental Table 6:** **The hyperparameter optimization range and results of TabNet**

| Hyper-parameter | Tuning range | Tuning result |
| --- | --- | --- |
| n_d | (8, 64) | 22 |
| n_steps | (3, 10) | 4 |
| gamma | (1, 2) | 1.89 |
| cat_emb_dim | (1, 3) | 2 |
| n_independent | (1, 5) | 4 |
| n_shared | (1, 5) | 1 |
| momentum | (0.001, 0.4) | 0.10 |
| mask_type | [“sparsemax”, “entmax”] | sparsemax |

**Supplemental Table 7:** **The hyperparameter optimization range and results of TabTransformer**

| Hyper-parameter | Tuning range | Tuning result |
| --- | --- | --- |
| dim | (32, 64, 128) | 32 |
| depth | (1, 2, 3, 6) | 3 |
| heads | (2, 4) | 4 |
| Weight_decay | (1e-6, 0.1) | 0.1 |
| learning_rate | (1e-6, 0.001) | 0.001 |
| dropout | (0, 0.1, 0.2, 0.3, 0.4, 0.5) | 0.4 |

**Supplemental Table 8:** **The hyperparameter optimization range and results of SAINT**

| Hyper-parameter | Tuning range | Tuning result |
| --- | --- | --- |
| dim | (32, 64, 128) | 32 |
| depth | (1, 2, 3, 6) | 1 |
| heads | (2, 4, 8) | 4 |
| dropout | (0, 0.1, 0.2, 0.3, 0.4, 0.5, 0.6, 0.7, 0.8) | 0.7 |

SAINT, Self-Attention and Intersample Attention Transformer.


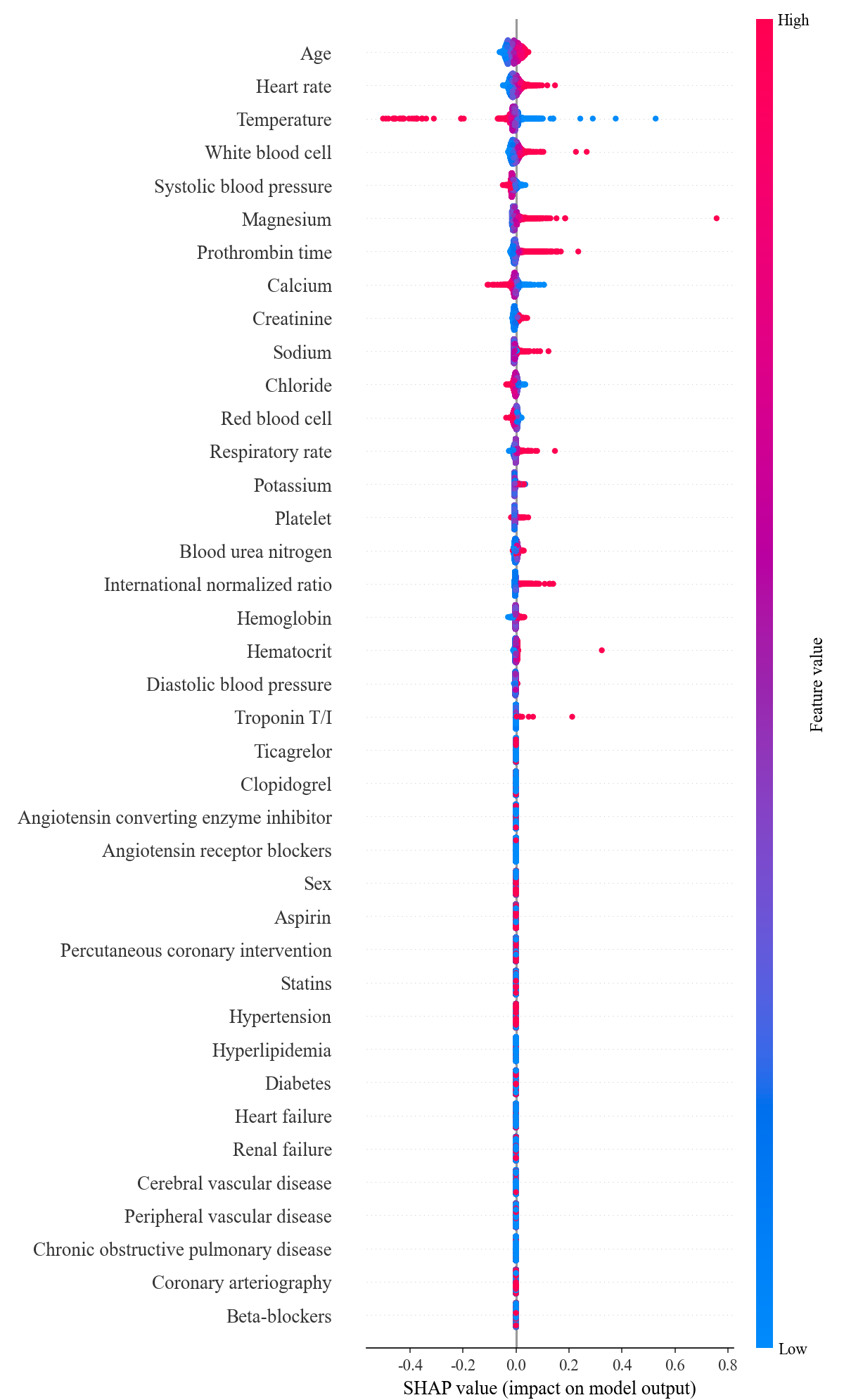


**Supplemental Figure 1:** The importance and contribution of the full features to the SAINT model.

**Supplemental Table 9:** The values of the evaluation metrics of the models in the internal test set (MIMIC-IV database and eICU-CRD were used for model training. The dataset of the Chongqing University Central Hospital was used for external validation).

|  | Balanced accuracy | Sensitivity | Specificity | Precision | F1 score | AUC |
| --- | --- | --- | --- | --- | --- | --- |
| LR | 0.76 (0.74, 0.78) | 0.80 (0.76, 0.83) | 0.72 (0.71, 0.74) | 0.23 (0.21, 0.25) | 0.35 (0.33, 0.38) | 0.83 (0.82, 0.85) |
| RF | 0.74 (0.72, 0.77) | 0.63 (0.59, 0.68) | 0.86 (0.85, 0.87) | 0.31 (0.28, 0.34) | 0.41 (0.38, 0.45) | 0.85 (0.83, 0.86) |
| XGBoost | 0.78 (0.76, 0.79) | 0.78 (0.75, 0.81) | 0.77 (0.76, 0.79) | 0.26 (0.24, 0.28) | 0.39 (0.36, 0.41) | 0.85 (0.84, 0.87) |
| LightGBM | 0.77 (0.75, 0.79) | 0.79 (0.75, 0.82) | 0.76 (0.75, 0.78) | 0.25 (0.23, 0.27) | 0.38 (0.35, 0.41) | 0.85 (0.84, 0.87) |
| CatBoost | 0.77 (0.76, 0.79) | 0.81 (0.77, 0.84) | 0.74 (0.73, 0.75) | 0.24 (0.22, 0.26) | 0.37 (0.34, 0.39) | 0.85 (0.83, 0.87) |
| MLP | 0.78 (0.77, 0.80) | 0.82 (0.79, 0.85) | 0.74 (0.73, 0.76) | 0.24 (0.23, 0.26) | 0.38 (0.35, 0.40) | 0.85 (0.84, 0.87) |
| TabNet | 0.73 (0.71, 0.75) | 0.72 (0.67, 0.76) | 0.75 (0.73, 0.76) | 0.22 (0.20, 0.24) | 0.34 (0.31, 0.36) | 0.82 (0.80, 0.83) |
| TabTransformer | 0.76 (0.74, 0.78) | 0.79 (0.75, 0.82) | 0.74 (0.72, 0.75) | 0.23 (0.21, 0.25) | 0.36 (0.33, 0.38) | 0.84 (0.82, 0.85) |
| SAINT | 0.78 (0.77, 0.80) | 0.86 (0.83, 0.89) | 0.71 (0.70, 0.72) | 0.23 (0.21, 0.25) | 0.36 (0.34, 0.39) | 0.86 (0.85, 0.88) |

**Supplemental Table 10:** The values of the evaluation metrics of the models in the external validation set (MIMIC-IV database and eICU-CRD were used for model training. The dataset of the Chongqing University Central Hospital was used for external validation).

|  | Balanced accuracy | Sensitivity | Specificity | Precision | F1 score | AUC |
| --- | --- | --- | --- | --- | --- | --- |
| LR | 0.76 (0.72, 0.79) | 0.72 (0.65, 0.79) | 0.79 (0.78, 0.81) | 0.20 (0.17, 0.24) | 0.32 (0.27, 0.36) | 0.84 (0.81, 0.87) |
| RF | 0.64 (0.61, 0.68) | 0.34 (0.27, 0.41) | 0.95 (0.94, 0.96) | 0.32 (0.26, 0.39) | 0.33 (0.27, 0.39) | 0.81 (0.77, 0.84) |
| XGBoost | 0.71 (0.67, 0.75) | 0.51 (0.43, 0.58) | 0.91 (0.90, 0.92) | 0.29 (0.24, 0.35) | 0.37 (0.31, 0.43) | 0.85 (0.83, 0.88) |
| LightGBM | 0.73 (0.69, 0.76) | 0.54 (0.46, 0.61) | 0.92 (0.90, 0.93) | 0.32 (0.26, 0.37) | 0.40 (0.34, 0.46) | 0.85 (0.82, 0.88) |
| CatBoost | 0.75 (0.71, 0.79) | 0.59 (0.52, 0.67) | 0.91 (0.90, 0.92) | 0.32 (0.27, 0.37) | 0.41 (0.36, 0.47) | 0.85 (0.82, 0.88) |
| MLP | 0.73 (0.69, 0.77) | 0.64 (0.56, 0.72) | 0.82 (0.81, 0.84) | 0.21 (0.17, 0.25) | 0.32 (0.27, 0.36) | 0.82 (0.79, 0.85) |
| TabNet | 0.73 (0.70, 0.77) | 0.60 (0.53, 0.68) | 0.87 (0.85, 0.88) | 0.25 (0.21, 0.29) | 0.35 (0.30, 0.40) | 0.84 (0.81, 0.87) |
| TabTransformer | 0.76 (0.73, 0.80) | 0.67 (0.60, 0.74) | 0.85 (0.84, 0.87) | 0.25 (0.21, 0.29) | 0.37 (0.32, 0.41) | 0.84 (0.81, 0.87) |
| SAINT | 0.78 (0.75, 0.81) | 0.72 (0.65, 0.78) | 0.84 (0.83, 0.86) | 0.25 (0.21, 0.29) | 0.37 (0.33, 0.42) | 0.86 (0.83, 0.89) |


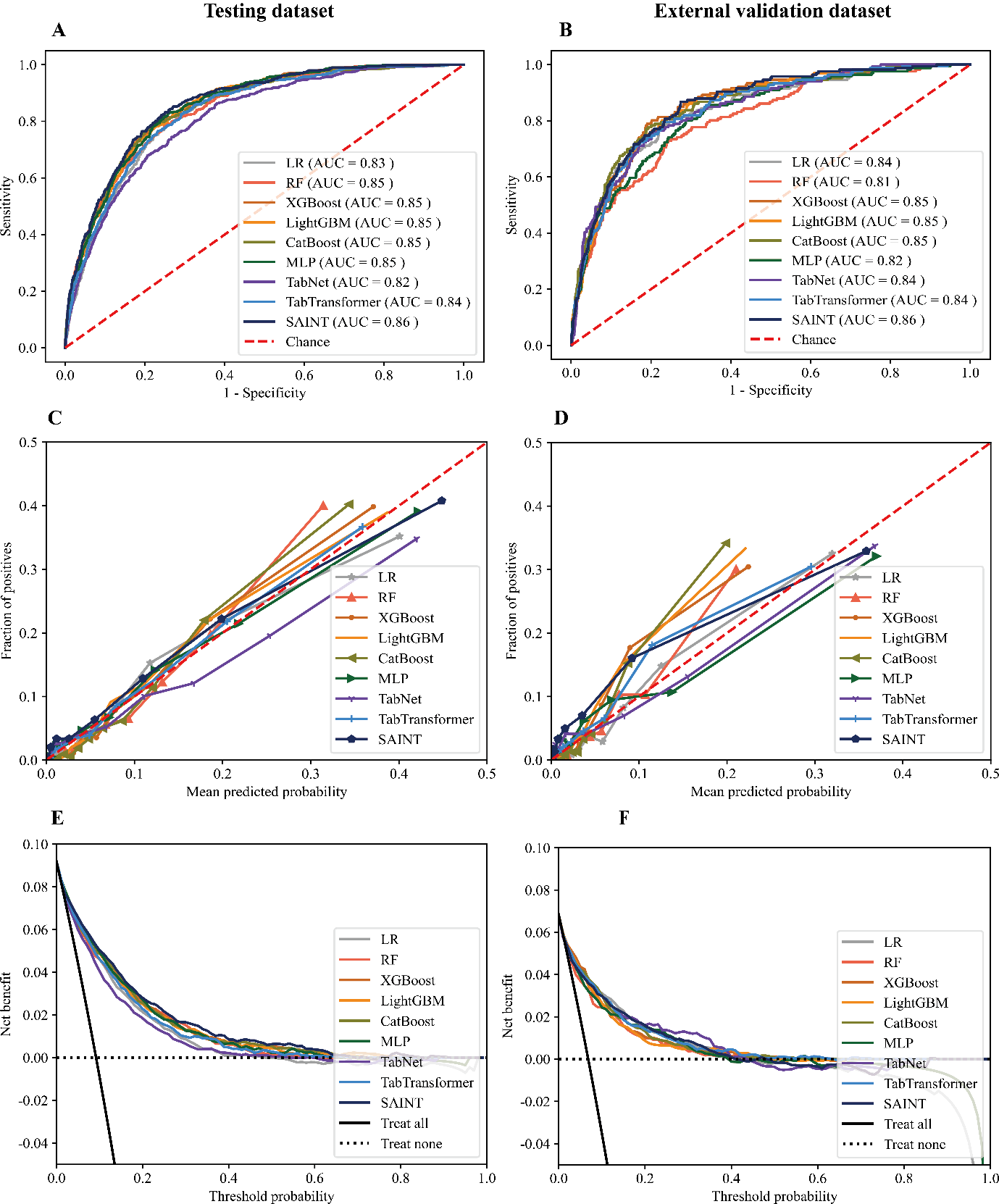


**Supplemental Figure 2:** Performance of the models in predicting in-hospital mortality of patients with AMI in the internal test set and external validation set. (A, B) AUCs of the models. (C, D) Calibration plots of the models. (E, F) Decision curves of the models. (MIMIC-IV database and eICU-CRD were used for model training. The dataset of the Chongqing University Central Hospital was used for external validation).

**Supplemental Table 11:** The values of the evaluation metrics of the models in the internal test set (The dataset of the Chongqing University Central Hospital was for model training. MIMIC-IV database and eICU-CRD were used for external validation).

|  | Balanced accuracy | Sensitivity | Specificity | Precision | F1 score | AUC |
| --- | --- | --- | --- | --- | --- | --- |
| LR | 0.79 (0.72, 0.85) | 0.74 (0.61, 0.86) | 0.84 (0.81, 0.87) | 0.25 (0.18, 0.33) | 0.38 (0.28, 0.47) | 0.89 (0.84, 0.93) |
| RF | 0.82 (0.76, 0.88) | 0.78 (0.66, 0.89) | 0.87 (0.84, 0.89) | 0.30 (0.22, 0.38) | 0.43 (0.34, 0.52) | 0.91 (0.86, 0.94) |
| XGBoost | 0.81 (0.75, 0.87) | 0.74 (0.61, 0.86) | 0.88 (0.86, 0.91) | 0.32 (0.23, 0.41) | 0.44 (0.34, 0.54) | 0.90 (0.87, 0.93) |
| LightGBM | 0.77 (0.71, 0.84) | 0.68 (0.55, 0.81) | 0.87 (0.84, 0.89) | 0.27 (0.20, 0.35) | 0.39 (0.30, 0.48) | 0.89 (0.85, 0.93) |
| CatBoost | 0.78 (0.72, 0.84) | 0.63 (0.50, 0.75) | 0.94 (0.92, 0.95) | 0.41 (0.31, 0.53) | 0.50 (0.39, 0.60) | 0.90 (0.86, 0.94) |
| MLP | 0.84 (0.78, 0.89) | 0.82 (0.71, 0.91) | 0.86 (0.83, 0.88) | 0.30 (0.22, 0.38) | 0.44 (0.35, 0.52) | 0.91 (0.86, 0.95) |
| TabNet | 0.77 (0.70, 0.83) | 0.76 (0.63, 0.88) | 0.77 (0.74, 0.80) | 0.20 (0.14, 0.25) | 0.31 (0.24, 0.38) | 0.85 (0.79, 0.90) |
| TabTransformer | 0.78 (0.72, 0.84) | 0.72 (0.60, 0.83) | 0.84 (0.82, 0.87) | 0.25 (0.19, 0.32) | 0.37 (0.29, 0.46) | 0.86 (0.79, 0.91) |
| SAINT | 0.86 (0.80, 0.91) | 0.84 (0.73, 0.93) | 0.87 (0.85, 0.90) | 0.33 (0.25, 0.41) | 0.48 (0.38, 0.56) | 0.91 (0.86, 0.95) |

**Supplemental Table 12:** The values of the evaluation metrics of the models in the external validation set (The dataset of the Chongqing University Central Hospital was used for model training. MIMIC-IV database and eICU-CRD were used for external validation).

|  | Balanced accuracy | Sensitivity | Specificity | Precision | F1 score | AUC |
| --- | --- | --- | --- | --- | --- | --- |
| LR | 0.68 (0.67, 0.69) | 0.83 (0.81, 0.85) | 0.53 (0.52, 0.54) | 0.15 (0.14, 0.16) | 0.26 (0.25, 0.27) | 0.76 (0.75, 0.78) |
| RF | 0.66 (0.65 0.66) | 0.93 (0.92, 0.94) | 0.38 (0.38, 0.39) | 0.13 (0.13, 0.14) | 0.23 (0.22, 0.24) | 0.74 (0.73, 0.75) |
| XGBoost | 0.70 (0.69, 0.71) | 0.73 (0.71, 0.75) | 0.68 (0.67, 0.68) | 0.19 (0.18, 0.20) | 0.30 (0.28, 0.31) | 0.77 (0.76, 0.78) |
| LightGBM | 0.69 (0.67, 0.70) | 0.69 (0.66, 0.71) | 0.69 (0.68, 0.69) | 0.18 (0.17, 0.19) | 0.29 (0.27, 0.30) | 0.75 (0.74, 0.76) |
| CatBoost | 0.68 (0.67, 0.70) | 0.73 (0.71, 0.75) | 0.64 (0.63, 0.64) | 0.17 (0.16, 0.18) | 0.28 (0.26, 0.29) | 0.75 (0.74, 0.76) |
| MLP | 0.69 (0.68, 0.70) | 0.83 (0.81, 0.84) | 0.56 (0.55, 0.56) | 0.16 (0.15, 0.17) | 0.27 (0.25, 0.28) | 0.76 (0.75, 0.78) |
| TabNet | 0.65 (0.64, 0.66) | 0.81 (0.79, 0.83) | 0.49 (0.48, 0.50) | 0.14 (0.13, 0.15) | 0.24 (0.23, 0.25) | 0.72 (0.70, 0.73) |
| TabTransformer | 0.67 (0.66, 0.68) | 0.88 (0.86, 0.89) | 0.46 (0.46, 0.47) | 0.14 (0.14, 0.15) | 0.24 (0.23, 0.26) | 0.74 (0.73, 0.75) |
| SAINT | 0.72 (0.70, 0.73) | 0.77 (0.75, 0.79) | 0.66 (0.65, 0.66) | 0.19 (0.18, 0.19) | 0.30 (0.29, 0.31) | 0.78 (0.77, 0.79) |


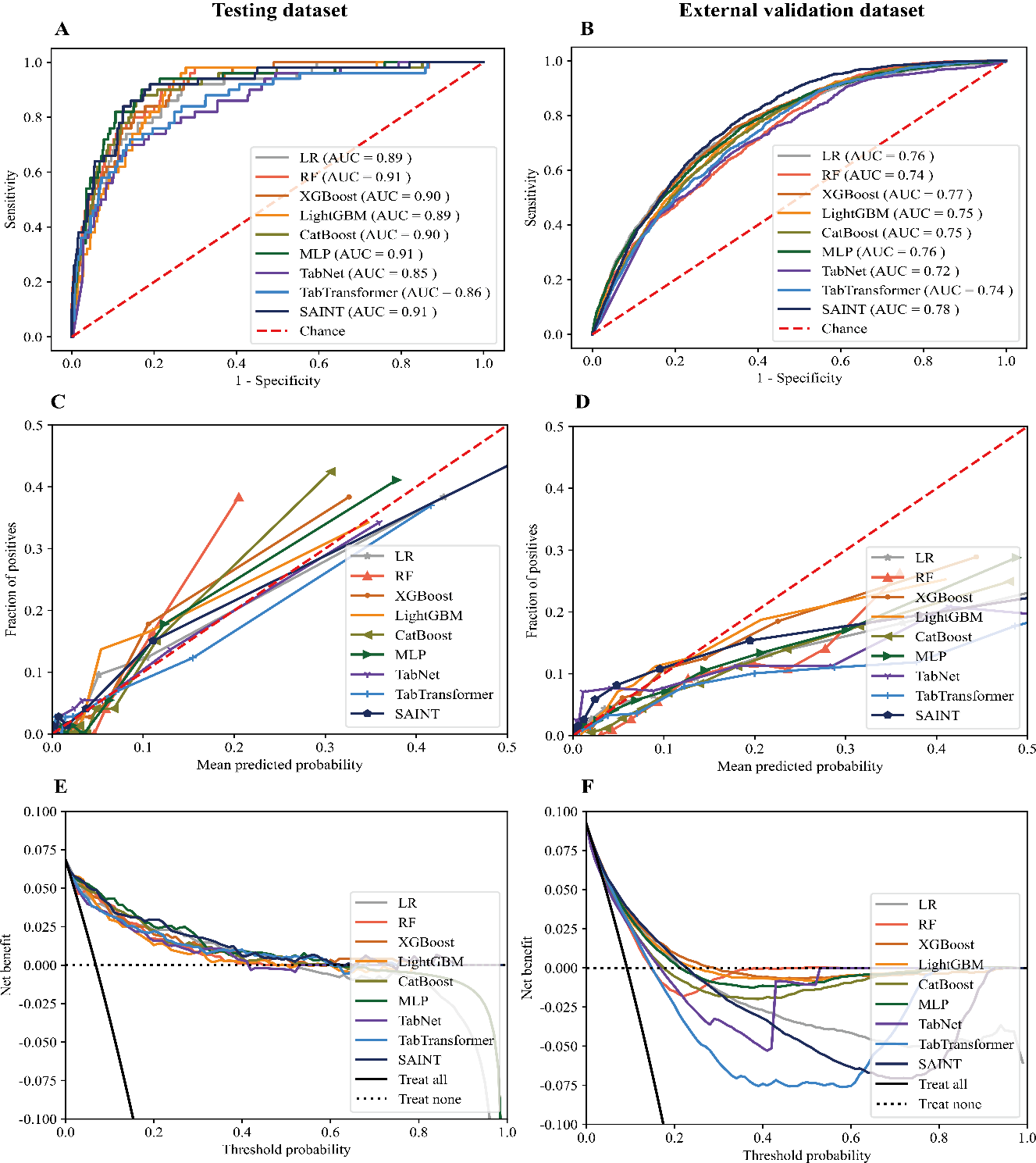
**Supplemental Figure 3:** Performance of the models in predicting in-hospital mortality of patients with AMI in the internal test set and external validation set. (A, B) AUCs of the models. (C, D) Calibration plots of the models. (E, F) Decision curves of the models. (MIMIC-IV database and eICU-CRD were used for model training. (The dataset of the Chongqing University Central Hospital was for model training. MIMIC-IV database and eICU-CRD were used for external validation).

**Supplemental Table 13:** The values of the evaluation metrics of the models in the external validation set (Only laboratory data obtained within two hours of admission were included. The dataset of the Chongqing University Central Hospital and eICU-CRD were used for model training. MIMIC-IV database was used for external validation).

|  | Balanced accuracy | Sensitivity | Specificity | Precision | F1 score | AUC |
| --- | --- | --- | --- | --- | --- | --- |
| LR | 0.71 (0.70, 0.72) | 0.87 (0.85, 0.89) | 0.55 (0.54, 0.56) | 0.16 (0.15, 0.17) | 0.27 (0.26, 0.29) | 0.79 (0.77, 0.80) |
| RF | 0.69 (0.67 0.70) | 0.82 (0.79, 0.84) | 0.55 (0.54, 0.56) | 0.16 (0.14, 0.17) | 0.26 (0.25, 0.28) | 0.76 (0.75, 0.78) |
| XGBoost | 0.69 (0.67, 0.70) | 0.68 (0.64, 0.71) | 0.70 (0.69, 0.71) | 0.18 (0.17, 0.20) | 0.29 (0.27, 0.31) | 0.76 (0.75, 0.78) |
| LightGBM | 0.68 (0.66, 0.69) | 0.72 (0.69, 0.75) | 0.63 (0.62, 0.64) | 0.16 (0.15, 0.18) | 0.27 (0.25, 0.28) | 0.74 (0.73, 0.76) |
| CatBoost | 0.70 (0.69, 0.72) | 0.65 (0.62, 0.68) | 0.75 (0.74, 0.76) | 0.21 (0.19, 0.23) | 0.32 (0.30, 0.34) | 0.78 (0.76, 0.79) |
| MLP | 0.71 (0.70, 0.72) | 0.88 (0.85, 0.90) | 0.54 (0.53, 0.56) | 0.16 (0.15, 0.17) | 0.27 (0.26, 0.29) | 0.79 (0.78, 0.80) |
| TabNet | 0.67 (0.66, 0.69) | 0.77 (0.74, 0.80) | 0.58 (0.57, 0.59) | 0.15 (0.14, 0.16) | 0.26 (0.24, 0.27) | 0.73 (0.72, 0.75) |
| TabTransformer | 0.72 (0.71, 0.73) | 0.88 (0.86, 0.91) | 0.56 (0.55, 0.57) | 0.17 (0.16, 0.18) | 0.28 (0.27, 0.30) | 0.80 (0.78, 0.81) |
| SAINT | 0.72 (0.70, 0.74) | 0.66 (0.63, 0.69) | 0.78 (0.77, 0.79) | 0.23 (0.22, 0.25) | 0.34 (0.32, 0.36) | 0.81 (0.80, 0.83) |


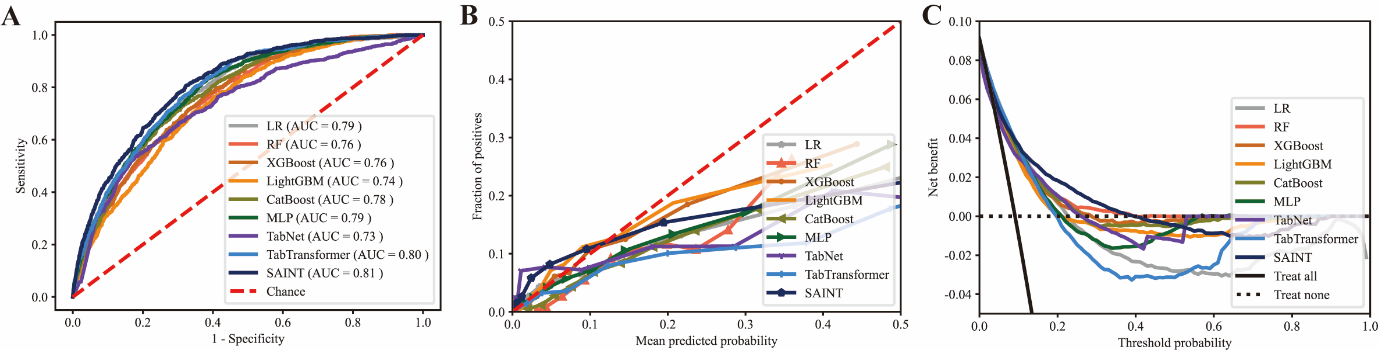


**Supplemental Figure 4:** Performance of the models in predicting in-hospital mortality of patients with AMI in the internal test set and external validation set. (A, B) AUCs of the models. (C, D) Calibration plots of the models. (E, F) Decision curves of the models. (MIMIC-IV database and eICU-CRD were used for model training. (The dataset of the Chongqing University Central Hospital was for model training. MIMIC-IV database and eICU-CRD were used for external validation).
